# Prevalence of adult sexual abuse in men with mental illness: Bayesian meta-analysis

**DOI:** 10.1192/bjo.2021.1069

**Published:** 2021-12-17

**Authors:** Milan Zarchev, Roos E. Ruijne, Cornelis L. Mulder, Astrid M. Kamperman

**Affiliations:** Epidemiological and Social Psychiatric Research Institute, Department of Psychiatry, Erasmus MC, the Netherlands

**Keywords:** Sexual abuse, victimisation, gender, abuse prevalence, review

## Abstract

**Background:**

Sexual abuse is a broad category of traumatic experiences that includes rape and any unwanted sexual contact with a body part or foreign object, whether penetrative, oral or otherwise. Although patients with mental illness have a higher risk of becoming victims of sexual abuse in adulthood, few studies investigate the proportion of male victims in this population. Their underrepresentation in research is a barrier to understanding the negative outcomes associated with sexual abuse in men.

**Aims:**

We estimated the prevalence of recent (past year) and adulthood sexual abuse perpetrated by any perpetrator and separately by intimate partners in males diagnosed with a mental illness.

**Method:**

To model the prevalences and heterogeneity arising from reports, we used Bayesian multilevel models. Prevalences were estimated for mixed-diagnosis, substance misuse, intellectual disability and post-traumatic stress disorder samples, and studies reporting specifically on intimate partner violence. This review was registered through PROSPERO (CRD42020169299)

**Results:**

Estimated adult sexual abuse was 5.3% (95% Credibility Interval 1.6–12.8%) for past-year abuse and 14.1% (95% Credibility Interval 7.3–22.4%) for abuse in adulthood. There was considerable heterogeneity of prevalence between studies and diagnosis groups.

**Conclusions:**

Our analyses show that the prevalence of sexual abuse of males diagnosed with a mental illness was much higher than for men in the general population. This has important implications regarding the proportion of undetected or untreated sexually abused men in clinical practice.

## Sexual abuse and its consequences

Sexual abuse is the most prominent risk factor associated with post-traumatic stress disorder (PTSD)^1,2^ and many other chronic DSM diagnoses.^3^ On average 10–19% of people are estimated to develop PTSD after a sexual abuse incident, according to the severity of abuse.^3^ In the general population, women are more often victims of sexual assault than men.^4,5^ However, in samples of people with physical and mental disabilities, equal numbers of men and women have been reported to be sexually victimised.^6,7^ Although all patients accessing psychiatric services have been found to be especially at risk of sexual abuse in adulthood,^8^ most research in this domain reports mainly or exclusively on female samples, thereby creating a gap in valid estimates for vulnerable male victims in a psychiatric setting. It was reported in a recent review on the association between sexual abuse and mental illness that 82% of all participants across 195 studies were female,^9^ and that 15 times more studies focused exclusively on women than on males or mixed samples.

This stark underrepresentation of males is problematic for two reasons. First, sexually victimised people with mental illness are at high risk of following a downward trajectory relating to their symptoms, coping and, ultimately, life expectancy. Not only is the severity of depression and anxiety worse in male patients who report a history of sexual abuse than in those who do not,^10^ such a history is also associated with acute intrusive thoughts, avoidant behaviours, poorer functioning and an overall general trend towards psychiatric morbidity.^11^ Men in general and clinical populations find it particularly challenging to disclose sexual abuse,^12^ which often leads to both lower self-esteem and to coping through substance misuse.^13^ As patients with mental illness are more likely to also report suicidal ideation and follow through with an attempt, sexual abuse emerges as a better predictive factor than any sociodemographic factor or other category of abuse.^14^ A robust association can thus be followed across the different lines of research, connecting sexual victimisation to worse psychosocial outcomes, self-medication coping, substance misuse and suicide attempts in male patients with mental illness. To assess the proportion of men vulnerable to this downward trajectory, our study was therefore intended to estimate the prevalence of recent and lifetime sexual abuse victimisation.

## The problem of heterogeneity

The second problem is that the lack of research on male sexual abuse makes it particularly difficult to establish basic epidemiological knowledge. When disparate studies report findings across a wide range of sample characteristics and research protocols, estimation becomes a significant challenge – which is certainly the case in this area. The literature on psychiatric victimisation shows substantial differences between outcome operationalisation, morbidity profiles and definitions of abuse.^15^ But despite the broad heterogeneity of their settings, all studies share a common investigative goal. Recognition of this common underlying goal indicates that the meta-analytic approach is a valid tool for establishing the overall prevalence trend, and, equally importantly, that this heterogeneity should also be explicitly modelled across studies.^16^ Bayesian methods offer the profitable opportunity to flexibly model heterogeneity across studies, obtaining stable estimates via mildly regularising priors where frequentist models might prove impossible to calculate.^17^

Variance in the prevalence of sexual abuse among psychiatric patients can arise from many sources. It is notable that research on sexual victimisation has been split between two types of study: those that focus exclusively on a familial or intimate relationship with the perpetrator, and those which do not distinguish how the victim was related to the perpetrator.^15^ Investigating intimate partner violence in particular is important, as it is characterised by a chronic, severe pattern of violence that does not allow the victim a safe space to recover.^18^ No review has investigated how the two strands of research on victimisation and intimate partner violence together inform the prevalence of sexual abuse among men diagnosed with mental illness. In its synthesis of the prevalence of sexual abuse across the literature, our study focuses particularly on taking into account the wide heterogeneity of results.

## Method

### Research aim

To estimate the prevalence of adult sexual abuse among males diagnosed with a mental illness, we investigated both the recent (i.e. past year) and adulthood prevalence of victimisation, reviewing the literature on the sexual abuse rates of male or mixed samples, including records in the literature on domestic violence. The current study follows PRISMA guidelines in reporting the meta-analytic synthesis.

### Inclusion criteria

We included male patients who met the definition of having a serious mental illness,^19^ namely a chronic psychiatric condition that disrupts daily functioning. Specifically, we searched for trauma-related disorders (for example PTSD), mood disorders (depression, bipolar), psychotic disorders (schizophrenia, psychosis), personality disorders (borderline personality disorder) and severe developmental intellectual disabilities, among general keywords for psychiatric diagnoses. We excluded articles that reported exclusively on children (under the age of 18), women, or participants drawn from the general population (i.e. men without mental illness). Neither did we consider reports on the perpetration of sexual assault, or reports focusing exclusively on childhood sexual abuse (CSA) as an outcome, as these lay outside the range of our review. If multiple studies reported on the same data, we included only the most detailed report.

### Outcome definition

Adult sexual abuse was defined as a broad category of experiences that included rape, any unwanted sexual contact or advance with a body part or foreign object (whether penetrative, oral or otherwise), that could be assessed on the basis either of standardised questionnaires (such as the Conflict Tactics Scale or Trauma Assessment for Adults), a structured clinical interview, or original questions enquiring directly into the patient's adult history. Intimate partner sexual abuse was defined and measured the same way, with the added dimension that the perpetrator was explicitly identified as a member of the victim's household.

### Search strategy and procedure

An electronic search was performed on 5 July 2020 using a predefined search algorithm in the following databases: Embase, Medline, Web of Science, Cochrane Central and Google Scholar. The search string used is presented in the Supplementary Data 1 available at https://doi.org/10.1192/bjo.2021.1069. The search target consisted of quantitative studies published since inception up to the search date on outcomes related to trauma and/or victimisation that include sexual abuse in samples of psychiatric in-patients or out-patients. Duplicates were removed from the resulting records. The remainder were then entered into Endnote (version 19.0). Before the start of screening, the study procedure was pre-registered with PROSPERO (ref. number CRD42020169299).

The following procedure was used to inspect titles and abstracts for mentions of sexual abuse and the characteristics of the sample. We looked specifically for quantitative reports on any sexual abuse of adult men with a DSM diagnosis. To be eligible for inclusion, studies had to present a proportion or prevalence of sexual abuse specifically for the male portion of the sample. Nationally representative samples were included if a psychiatric subsample was reported on separately. If possible, raw data was extracted from tables, charts or text and was converted to the relevant proportion. If we suspected that data was available even if no explicit prevalence was reported (for example, if gender groups or types of trauma were combined), the authors were contacted via email to enquire after the proportion. Screening was performed by the first (M.Z.) and second author (R.E.R.) independently. Discrepancies between these authors were discussed with a third author (A.M.K.) until consensus was reached.

### Data extraction

Data extraction began 5 July 2020 and concluded 3 August 2020. We extracted sample characteristics such as age group, ethnic majority, country of data collection, adulthood or past year of sexual abuse, and perpetrator of assault where available. Methodological information about the study was also recorded, including sampling strategy, instrument used, response rate, study design and study aims. Additionally, the implementation of the instrument was coded as follows: as ‘face-to-face’ when a clinician had administered the interview or questionnaire; or as ‘self-report’ when the participant had completed the questionnaire himself or had used computer-assisted methods. The full text of each report was inspected by the first author (M.Z.) and by the second author (R.E.R.), independently.

### Data quality

To assess the quality of studies, a form was adapted from the checklist of Hoy et al (2012) on the risk of bias in prevalence studies.^20^ Each study was scored on eight items relating to the following: data collection, non-responder proportion, representativeness and selection of samples, and outcome definition and psychometric properties of instrument. Per study, a score of 0, 1 or 2 was assigned to each item respectively to indicate no risk of bias, an ambivalent risk or a high risk. A dichotomous classification of good/low quality was assigned to each study according to whether three or more items were classified as high risk.

### Statistical analysis

A three-level random-intercept model was used to estimate the overall prevalence (level 1), prevalence of each diagnosis group (for example studies focusing on individuals who misused substances, on individuals with PTSD, mixed samples; level 2) and the prevalence of each study (level 3). This approach was preferred because of its flexibility in estimating an individual prevalence for each study and combining these in a way that prevents smaller studies from being completely outweighed by larger samples. This is valid under the realistic assumption that the individual studies do not all estimate one ‘true’ prevalence, but instead contribute to an overall random distribution of coexisting prevalences.

The likelihood of the data was modelled using a binomial distribution. An intercept-only model was used to estimate the sexual abuse prevalence. The logit-transformed intercept (α_i_) was allowed to vary for each study and each diagnosis group by following a normal distribution with a mean (μ), approximating the overall population prevalence and having two variance components: a between-group variance component (between-group τ) reflecting differences between the different diagnosis groups studies report on; and a between-study variance component (between-study τ), reflecting the between-study variance. The latter τ components are difficult to interpret in isolation, but useful in relative comparisons with each other as they indicate how much heterogeneity is present on each level of the model. In terms of heterogeneity, we also report on the *I*^2^ metric and interpret it using modern guidelines.^21^

We also used fixed-effect meta-regression methods for variables extracted from studies that had few missing data points – in this case, country; sampling strategy (random versus convenience); questionnaire used (validated questionnaire, clinical interview, original questions); instrument implementation (face-to-face versus self-report); mean sample age and year of publication. For the mean sample age and year of publication regression, we used a classical linear meta-regression, whereas for the categorical predictors, we compared study groups using a univariate regression method closely resembling ANOVA methods.^22^ We report on estimated differences in prevalence between categories based on this method. We use these univariate meta-regression instruments to provide a maximally intuitive idea of where between-study difference lie at the cost of opting out of more sophisticated multilevel multivariable models.

We report on estimates of prevalence for each country using a two-level random-effects model, where country instead of individual studies were used as the higher level. We chose this random-effects over fixed-effects model, in order to stably estimate the prevalence of countries represented by even a single study. Finally, as a sensitivity analysis, studies of low quality were removed to check whether using only high- quality studies produced a higher or lower prevalence.

To estimate the model parameters, we opted for a fully Bayesian approach. For the current research question, this method of inference has several advantages. It allows uncertainty to be modelled and propagated across all parameters in the model without relying on asymptotic standard errors^23^. Additionally, the posterior distribution produced in Bayesian analysis allows for an intuitive interpretation in terms of probability in contrast to classical confidence intervals. Regarding uncertainty around estimates, we report here on highest density intervals, where 95% of the probabilities are amassed.

Bayesian inference requires the specification of prior beliefs on the parameters to be estimated. Updating the priors with available data is conceptually the core procedure behind constructing the posterior distribution. We supplied vaguely informative priors that do not give credibility to impossibly extreme values and are easily overwhelmed by empirical data.^16^ We expected 95% of the prevalence probability to fall between 2% and 98% (μ ~ Normal(0,2)), and a half-Cauchy distribution was chosen for the between-study deviation estimate (τ ~ half-Cauchy(0,1)), which described non-zero positive values. The half-Cauchy distribution is considered particularly appropriate for modelling between-study variance in meta-analyses.^24^ Sensitivity analyses were conducted for the parameters used in the intercept prior.

For all inferential statistics, we used the R package brms (version 2.11), which is an interface for the probabilistic programming language and compiler STAN.^25,26^ To assess goodness-of-fit, we used posterior predictive checks to generate new data from the model and to assess how well the generated data follow the data that had actually been collected. Model convergence is assessed by visually inspecting trace and autocorrelation plots. Additionally, Rhat values of each parameter estimates were checked for values higher than 1.1, which are used to indicate poor convergence.^25^ We did not encounter convergence issues. Models were compared using Widely Applicable Information Criteria (WAIC) and Leave-One-Out (LOO) cross-validation, whose interpretation is close to the classic Bayesian and Akaike information criteria (lower values correspond to better fit), but whose computational properties are better.^27^

## Results

### Study selection

**Fig. 1:**
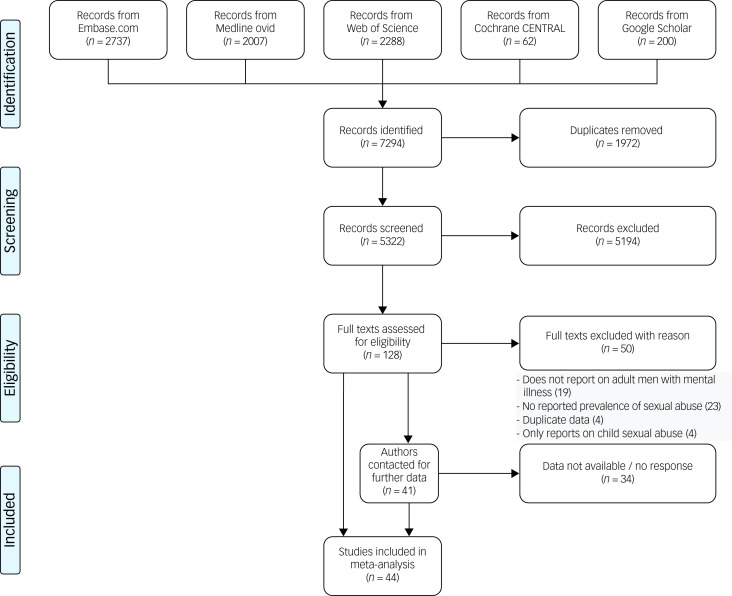
Flow chart for the selection process behind the final studies included in the current meta-analysis.

Figure 1 presents the selection process for the studies included in the current analysis. From all sources combined we identified 7294 records. Of those, we found 37 records that met the inclusion criteria. We also identified 41 studies that potentially met the criteria, but did not explicitly report on the prevalence we needed. After contacting the authors of those studies, seven provided us with the data we needed. Thus, there were 44 records in total eligible for the current meta-analysis. Interrater reliability was considered substantial (raw interrater agreement was 90.6%; Cohen's κ 0.79 (95% CI 0.68–0.90).

### Study characteristics

A total of 44 studies were included in the meta-analysis, two of which contributed two data points each. Samples consisted primarily of mixed ethnicities (63%). The unweighted mean age across samples was 38.5 years. Although a majority of studies were published after 2005 (65%), only 43% of the data on which they reported had been collected after 2005. Overwhelmingly, studies reported on high-income Western countries, with the exception of five studies, one reporting on Egypt,^28^ one on India^29^ and one on Taiwan^30^ and two studies on Brazil.^31,32^ With regard to the main diagnosis, 15 studies reported on samples with a mixture of DSM diagnoses (hereafter referred to as mixed-diagnosis samples; six contained information on past-year abuse, 11 on adulthood abuse), 17 reported exclusively on adulthood sexual abuse in substance-misusing samples; three (two past year, three adulthood) on psychosis, three on past-year sexual abuse in intellectually disabled samples; and two on PTSD (one past year, two adulthood abuse). Additionally, six studies reported on mixed-diagnosis samples in which sexual abuse had been perpetrated by intimate partners (three on past-year abuse, four on adulthood). In total, substantially more studies reported on adulthood prevalence of sexual abuse (*k* = 40) than on past-year incidence (*k* = 12).

All studies involved a clinician or trained specialist in assigning psychiatric classification, except for one national survey that employed self-report.^33^ We found that prevalence estimates changed by less than 1% when excluding this single study, so we proceeded with including it in our results. Although data was collected on the proportion of DSM diagnoses and comorbidities in each sample, we eventually found that such a level of detail was provided by only a fraction of studies.

Sample size covered a wide range, the median was 175 participants and the largest study included 33 236 participants. The total number of participants across all studies was 45 172. Supplementary Table 1 provides a full list of the included studies. Four studies reported on odds ratio comparisons between sexual abuse prevalence in men with mental illness versus men in the general population.

### Risk of bias

**Fig. 2:**
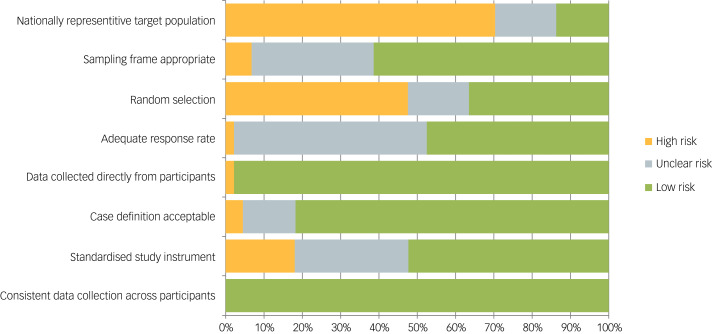
Quality assessment for the studies included in the current meta-analysis.

The scores of a small majority of studies (*k* = 27, 61%) indicated a low risk of bias. Figure 2 presents the proportions of bias across quality items. As might be expected, few of the studies reported on nationally representative samples. Similarly, over half the studies either relied on some sort of convenience sampling, or were unclear about their selection method. Around 30% were unclear on the sampling frame and the validity of the instrument used. Nearly 50% did not provide a response rate, in many cases due presumably to the non-random nature of the sample. However, nearly all studies had acceptable data collection procedures, and also defined outcomes appropriately.

### Model selection

For comparison with the main random-effects model, a fixed-effect model was initially fitted to the past-year and adulthood prevalence studies. This produced prevalences of 4.11% (95% Credibility Interval 3.46–4.80%) for past-year sexual abuse and 9.85% (95% Credibility Interval 9.57–10.14%) for adult sexual abuse. Although these estimates were considerably more precise than the random-effects model we fitted next, the fixed-effects specification was a relatively poorer fit to the data. For the past- year and adulthood fits, WAIC and the LOO cross-validation statistics were both over half those in the random-effects model. WAIC and LOO values for all models are presented in Supplementary Table 2. It was also shown by posterior predictive checks of the way in which the models estimated the overall mean – and especially the means for the individual studies – that the random-effects model produced predictions that were much closer to the empirical observation. On the basis of these tests, we chose to use the random-effects model for reporting further estimates. The posterior predictive check showed that the random-effects model captured the observed variation between studies and populations well within the credibility intervals of the estimates (Supplementary Fig. 1).

### Synthesis of prevalence

**Fig. 3:**
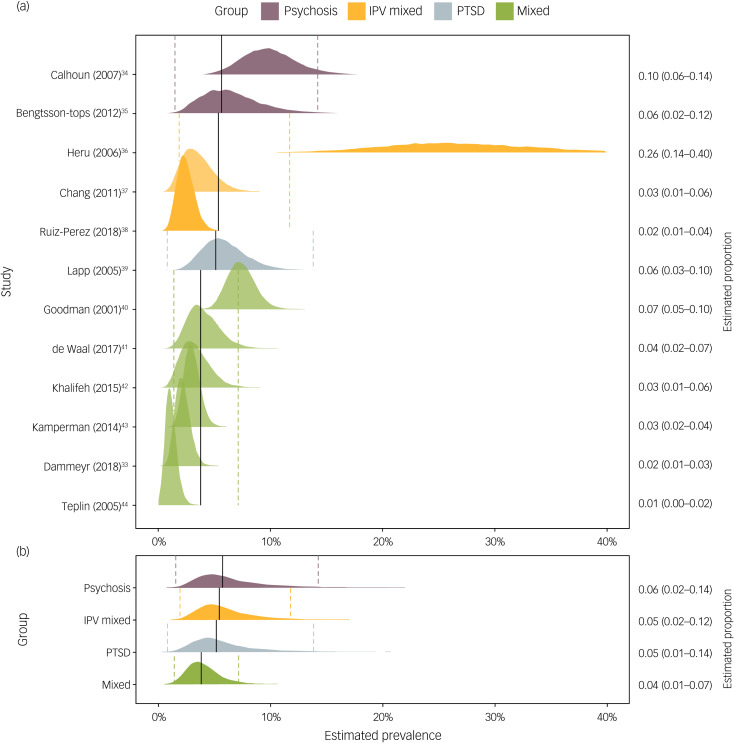
Individual study (a) and group-level (b) estimates of past-year sexual abuse in psychiatric patients. Presented in text on right column proportion estimates and 95% credibility intervals. Vertical solid lines indicate mean group-level estimates, dashed lines corresponding 95% credibility intervals. Weights for the analysis were obtained from the random effects produced by the model. IPV, intimate partner violence; PTSD, post-traumatic stress disorder.

Past-year group and study estimates can be found in the forest plot in Fig. 3. After inclusion of all the studies in the analysis, estimated overall prevalence was 5.3% (1.6–12.8%) for past-year sexual abuse and 14.1% (95% Credibility Interval 7.3–22.4%) for adulthood sexual abuse. Looking at past-year prevalences, it was estimated that psychosis samples reported the most sexual abuse with a 5.7% estimated prevalence. Next, studies reporting on intimate partner violence in mixed samples produced an estimate of 5.4% (95% Credibility Interval 1.9–11.8%). A single study on past-year sexual abuse in PTSD samples produced an estimate of 5.1% (95% Credibility Interval 0.01–13.9%). Finally, mixed-diagnosis reports produced an estimate of 3.8% (95% Credibility Interval 1.4–7.2%).

**Fig. 4:**
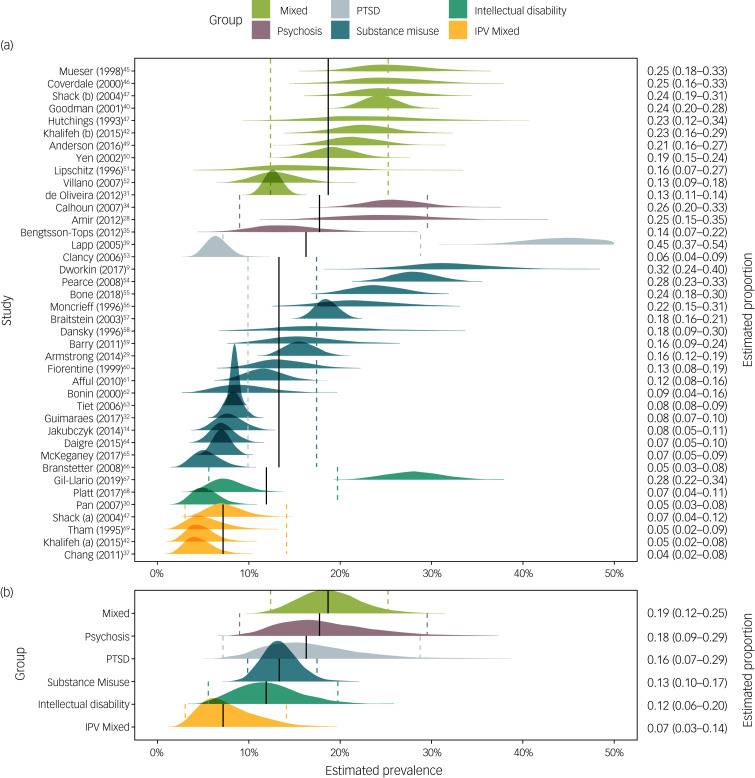
Individual study (a) and group-level (b) estimates of the prevalence of adult sexual abuse in psychiatric patients. Presented in text on right colum proportion estimates and 95% credibility intervals. Vertical solid lines indicate mean group-level estimates, dashed lines corresponding 95% credibility intervals. Weights for the analysis were obtained from the random effects produced by the model. IPV, Intimate partner violence; PTSD, post-traumatic stress disorder.

For adulthood sexual abuse, study and group estimates can be found in Fig. 4. The highest prevalence was estimated in the mixed-diagnosis group at 18.7% (95% Credibility Interval 12.4–25.2%), followed by the psychosis group at 17.7% (9.0–29.4%). Prevalence among those with PTSD was estimated at 16.3% (95% Credibility Interval 7.2–28.7%), but the estimate was based on two studies reporting very different results (5.9% and 19.5%), therefore the estimate reported here does not capture either of the two empirical findings. The substance misuse group produced an estimated prevalence of 13.3% ((95% Credibility Interval 10.0–17.4%) followed by the intellectual disability group with an estimate of 11.8%.

**Fig. 5:**
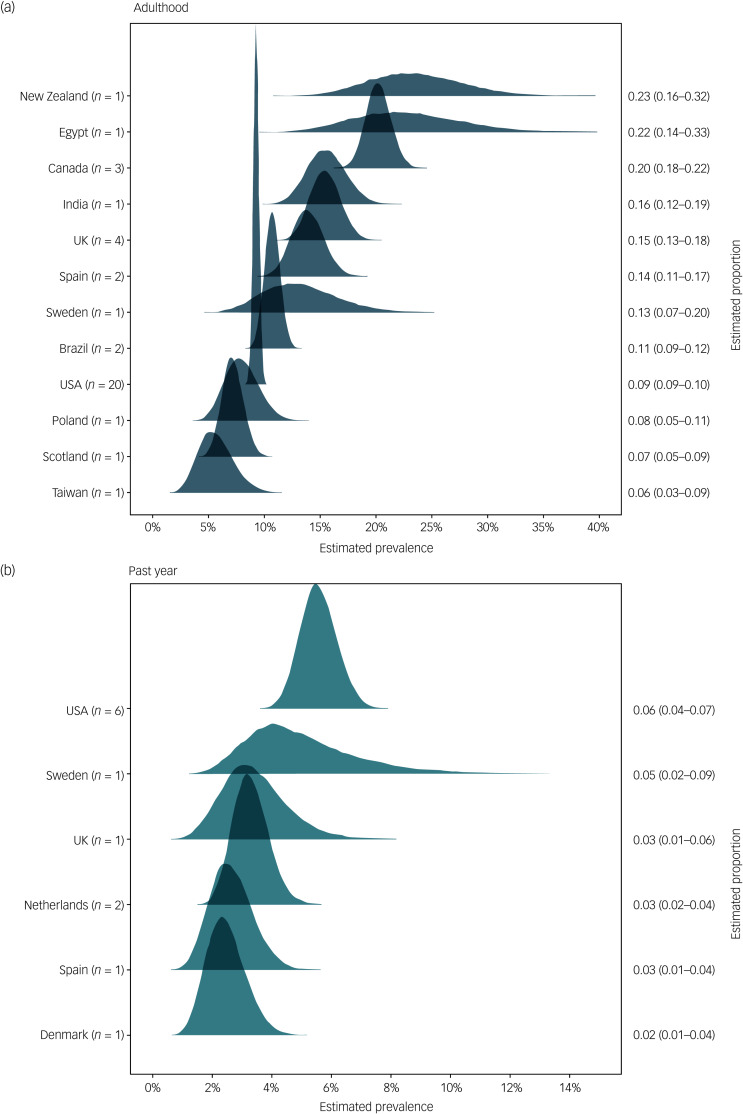
Estimated overall prevalence of sexual abuse for men with mental illness by country for (a) adulthood sexual abuse and (b) past-year sexual abuse.

Finally, intimate partner violence from mixed-diagnosis samples was estimated at 7.2% (95% Credibility Interval 3.0–14.1%). This last group estimate should be noted is higher than the estimates reported from the majority (three out of four) individual studies reporting on lower estimates of intimate partner violence. This is because of the desirable property of shrinkage in multilevel models.^22^ Briefly, this indicates that the intimate partner violence group estimate is somewhat uncertain because of the low sample size studies it is based on and therefore it is shrunk towards the global mean.

As we had expected, the between-study variance was relatively high: between-study τ = 0.99 (95% Credibility Interval 0.52–1.53) for past-year abuse and 0.70 (95% Credibility Interval 0.52–0.89) for adulthood abuse. These were higher than the between-group τ variance that was 0.57 (95% Credibility Interval 0.00–1.50) for past year and 0.60 (95% Credibility Interval 0.03–1.21) for adulthood. The posterior estimates for the variance parameters are visualised in Supplementary Fig. 2. These variance parameters indicate that there are more differences in prevalence between study than between different types of populations sampled (for example between individuals who misuse substances and patients with psychosis), especially so for past-year reports. *I*^2^ estimates were also high at 82.1% for the past-year studies and 91.9% for adulthood studies. This indicates that even if the current studies provided highly powered and precise estimate (e.g. by including many more participants in their study sample), we would still expect to find substantial relative differences in reported prevalences above and beyond those arising from sampling error. We turn to meta-regression methods to investigate the sources of between-study variance.

### Other results

#### Meta-regression

A fixed-effect meta-regression was constructed to investigate the overall effect of study characteristics. Information was widely available on mean sample age, year of data collection, screening instrument used, sampling design and implementation of the instrument. Sampling design referred to whether a convenience sample was used (*k* = 28) or participants were randomly enrolled (*k* = 16). As nearly every study had used a different questionnaire to screen sexual abuse, questionnaires were categorised as validated questionnaires (*k* = 23), non-validated original questions (*k* = 10) or standard clinical interviews (*k* = 11). The implementation of the instrument was coded as self-completed by participants (*k* = 7), or completed by an interviewer during a face-to-face dialogue (*k* = 37). Meta-regression analyses were conducted on both past-year and adulthood data. The posterior results for all parameters are presented in Supplementary Fig. 3.

We first investigated whether the prevalence produced by face-to-face reports was different from that produced by self-completed reports. This showed that face-to-face studies produced a prevalence for adulthood reporting that was considerably lower (9.6%) than that produced by self-report studies (21.1%), a difference of Δ11.5% (8.67–14.3%). Conversely, for past-year reports, the prevalence obtained by face-to-face studies (4.5%) was higher than that obtained by self-report (2.3%), a smaller difference of Δ2.2% (95% Credibility Interval 0.1–4%). When the questions pertained to adulthood abuse, self-completed reports thus produced higher prevalences than face-to-face interviews, but lower prevalences when the questions pertained to past-year abuse.

Next, we compared prevalences on the basis of the measurement instrument used. The lowest pooled estimate for the adulthood period was produced by the validated questionnaire studies (0.9%), followed by the structured clinical interviews (13.4%), followed by the highest estimate for original questions (15.3%). The credibility intervals for all differences were above zero, starting with Δ6.2% (95% Credibility Interval 4.9−7.6%) for the original – validated questionnaires comparison, Δ4.3% (95% Credibility Interval 3.1–5.5%) for the clinical interview – validated questionnaire comparison and finally Δ2% (95% Credibility Interval 0.2–3.8%). For the past-year period, validated questionnaires instead reported higher prevalences (4.8%) than original questions (2.1%), a difference of Δ2.7% (95% Credibility Interval 1.4–3.9%). No studies had used clinical interviews in the past-year period. It appears that the more rigorous methods produced lower estimates for the adulthood period and higher for the past-year period.

Initial comparisons of convenience-sample studies and random-sample studies for the adulthood period showed a higher prevalence in the random-sample studies. To understand this counterintuitive finding, we added a separate fixed estimate for each diagnosis subgroup. This analysis showed a clear pattern for all subgroups: random sampling produced lower estimates than convenience sampling, although a relatively low prevalence was reported by a single very large convenience sample study in the substance misuse subgroup. Repeating this type of subgroup analysis revealed no similar contrasting trends in the previous instrument and implementation models. For the sake of parsimony, we removed the large study with considerable weight, and report on overall estimates rather than individual subgroup differences. This resulted in a Δ1% difference between convenience (14.2%) and random samples (15.2%) for the adulthood period. For the past-year period, random samples produced considerably lower estimates (3.3%) than convenience samples (8.7%), a difference of Δ5.5% (95% Credibility Interval 3.1–8.2%).

Finally, a univariate regression showed no evidence for effect of year of publication for adulthood abuse, but a negative association for past-year abuse (odds ratio (OR) = 0.94, 95% Credibility Interval 0.91– 0.96). There was no evidence that mean sample age was associated with prevalences in past-year abuse, but there was a negative association for adulthood prevalences (OR = 0.96, 95% Credibility Interval 0.95– 0.97).

#### Prevalence by country

A random-intercept model was fitted for past-year and adulthood sexual abuse, thus allowing the intercept to vary by country of study. Figure 4 presents the ranked results, which show that past-year estimates were generally more precise than adulthood estimates. The estimates for countries with few participants across studies were both uncertain and difficult to interpret, especially those for New Zealand, Egypt and Sweden. With regard to relative rank, past-year sexual abuse was the most prevalent in patients with mental illness in the USA (6%) and the least prevalent in Denmark (2%). Divergent results were obtained for the prevalence of adulthood sexual abuse, in that New Zealand (23%) and Egypt (22%) ranked highest, whereas Taiwan (6%) and Scotland (7%) were lowest. As both of the latter estimates were relatively imprecise, it is certain that the empirical point estimate was shrunk considerably towards the global average.

#### Comparison with the general population

To compare prevalence rates, we collected odds ratios from studies that contained a general-population control group. This was reported *ad hoc*, as we expected no more than a few studies to include a control group. Two studies reported on adulthood sexual abuse and two on past-year abuse. These odds ratios are presented in Table 1; odds ratios were calculated manually from one study that reported only on summary statistics and *P*-values.

**Table 1:** Odds ratios comparing the samples with mental illness with the general-population control groups

Study	Comparison sample (period)	OR (95% CI)^a^	*n* participants in general population
Afful et al (2010)^61^	Substance misuse (adulthood)	1.8 (0.9–3.7)	216
Khalifeh et al (2015)^42^	Mixed-diagnosis (adulthood)	9.3 (6.3–13.7)	10 317
Kamperman et al (2014)^43^	Mixed-diagnosis (past year)	6.3 (3.6–10.3)	38 227
Dammeyer & Chapman (2018)^33^	Mixed-diagnosis (past year)	3.7 (1.8–7.5)^b^	12 707

a. Comparison sample versus general population.

b. Calculated manually from summary statistics on group membership.

#### Diagnostic and sensitivity analyses

Trace plots for the four models appeared to be well mixed, and showed little to no divergence. This was further evidenced by Rhat estimates, which neared or equalled 1 for all parameters. On visual inspection, autocorrelation plots revealed no autocorellated parameters.

A sensitivity analysis was performed by first removing the low-quality studies (*k* = 17). The overall prevalence remained changed by less than a percentage point for the past-year estimate. The adulthood estimate was increased from 14.1% (95% Credibility Interval 7.3–22.4%) to 15.3% (95% Credibility Interval 6.5–29.6%). Further sensitivity analyses were then conducted for the priors of the prevalence model. Changing the parameters for the intercept prior with more informative alternatives (normal distributions with a sigma parameter of 1 or 0.5) produced no or only minimal variation in the posterior distribution (a change of less than a percentage point for past-year and adulthood models). Likewise, switching the half-Cauchy prior on the variance estimate for an alternative gamma distribution did not meaningfully alter the between-study variance posterior. We conclude that the priors were very weakly regularising, only preventing extreme values from being estimated.

## Discussion

### Main findings

Our meta-analysis found high estimated prevalences of sexual abuse across all the groups of patients with SMI investigated. The highest adulthood pooled prevalence (19%) was reported by the mixed-sample studies (two or more clinical diagnoses present in sample across patients), followed by studies in the psychosis samples (18%), substance misuse samples (13%) and intellectual disability samples (12%). The lowest estimated adulthood prevalence (7%) was for sexual abuse in studies investigating perpetration by an intimate partner in the mixed-samples studies.

Past-year sexual abuse was estimated at 5%, with highest prevalences reported in studies with psychosis samples (6%), followed by mixed samples (5%). There were not enough studies to robustly estimate the prevalence of sexual abuse in those with PTSD beyond the observed data.

Considerable between-study variance was a pervasive feature across all analyses. We found that a portion of this variance was because of the characteristics of the study design (such as lower estimates were found in random sampling versus higher in convenience sampling), and also because of the country in which the data had been collected.

Clear differences are shown by comparison of our estimates and the prevalences reported in non-clinical groups. For men, adult sexual abuse ranges from 1% to 7% in general-population samples.^4,70–72^ This difference was similarly reflected by the *post hoc* general-population analysis we conducted, showing increased odds of sexual victimisation in men belonging to the psychiatric patient group. As such, the risks associated with being a psychiatric patient could translate to a three-times higher risk of sexual abuse. In contrast, intimate partner violence was estimated to be somewhat similar to that reported for men in the general populations. Estimates for sexual abuse of non-psychiatric patients range between 0.5 and 5%.^42,73,74^ Considering the 7%-point estimate for psychiatric patients in the current sample, it is clear that defining intimate partner sexual abuse as ‘when a woman experiences some kind of violence’^75^ is an archaic description. Men can experience violence at home as well. For a measured discussion of gender and violence in psychiatric populations, see Khalifeh & Dean.^15^

Additionally, men with mental illness in the present studies did not seem to be at a higher risk of sexual abuse perpetrated by an intimate partner than men in the general population. Broadly, in adulthood men are more likely to be victims of sexual abuse perpetrated by strangers compared with the opposite pattern observed in women.^71^ This heightened risk of assault by strangers explains plausibly why our analysis could detect a considerable difference only when the focus lay on non-intimate partner violence sexual abuse rather than on the relatively rarer intimate partner violence sexual abuse incidents.

### Interpretation of our findings relating to substance misuse

A striking finding was that the estimated prevalence of sexual abuse in mixed-diagnosis samples was 7% higher than in substance-misusing samples. All studies relied on recollection of sexual abuse, therefore it is theoretically possible the mixed sample and those samples with people with psychosis inflated their reporting because of disordered thinking. Research focusing on reliability of crime reports from people with SMI, however, point out that self-reports are generally stable over time and in fact men in that population are more likely to underreport rather than overreport sexual abuse.^76^ Even more puzzling, the relatively lower prevalence in people with substance misuse is not entirely consistent either with the robust and long-established link between sexual violence and high substance misuse as a common coping strategy.^77^ People with substance misuse are more likely to find themselves in a sheltered housing environment, and data supports that those conditions are more protective against victimisation in psychiatric populations compared with living independently.^78^ However, the finding is likely also related to the between-study variability in this group of studies. Not only was this variance highest across all groups, with individual report estimates ranging from 5% to 32%, the studies that reported the highest prevalences were also those that reported on people with substance misuse. To explain this considerable heterogeneity, we also draw attention to the fact that the studies included in the analysis differed considerably with regard to the specific drug addictions (for example stimulants or depressants) that afflicted the majority of the sample. Differences in drug use are reported to result in differences in victimisation: for example, addiction to alcohol and/or cannabinoids carry a much higher risk of victimisation than addiction to sedatives.^79^ A caveat of our analysis is thus that the resulting estimate of the prevalence for people with substance misuse may underestimate victimisation in individuals with alcohol misuse, but overestimate that of others such as people with tranquilliser misuse. However, the prevalence reported here is also the best estimate of sexual abuse in people with substance misuse overall, as it incorporates both information and uncertainty from a wide pool of related literature.

### CSA

The scope of the current meta-analysis did not include discussion of CSA, despite the severe consequences on mental health that follow such type of trauma.^80^ Although the debilitating impact of CSA can be similar to that of sexual abuse during adulthood such as adopting substance use as a coping mechanism or increased psychopathology, there are also important differences.^81^ Namely, wide-ranging developmental problems are by definition within the domain of CSA, reflected deeply in neurological, hormonal and epigenetic markers.^82^ As such, sexual abuse during childhood should be of major concern to the study of trauma in patients with SMI. That area of research, however, presents its own issues of definition, measurement and study design when collecting data on CSA, especially in the context of SMI samples.^83^ Sensitive expert attention should be paid to those idiosyncratic issues when synthesising the immense CSA literature, which lie far beyond the scope of the current review. Had studies asking specifically about CSA been included, lifetime prevalences would be even higher than the ones reported here.

Unfortunately, research is equally lacking on CSA of men with SMI as with adult sexual abuse. It is up to future work to synthesise exactly what the prevalence of male CSA abuse in men with SMI is. Insights from the extensive CSA literature can be useful, however, in assessing the limitations of research on adult sexual abuse. Importantly, there is evidence men do not disclose CSA for decades, over 30 years on average after the event happened.^84^ Eventual disclosure to a mental health professional however, was linked with better health and functional outcomes. There is no evidence to contradict either finding for adult sexual abuse. As such, this provides further evidence that the already worryingly high prevalences reported in the current synthesis are potentially underestimates of how often men with SMI become victims of sexual abuse, especially the past-year estimate where participants might defer to disclose traumatic events until years later. Conversely, when barriers to disclosure are surmounted and medical professionals are involved, there is optimistic evidence that health outcomes, mental or otherwise, can be improved.

### Study quality

Our meta-analysis addressed a narrowly defined target group of men with mental illness who had been victims of sexual abuse. Despite the specific inclusion criteria, the studies we identified were substantially heterogeneous, both in their methodology and their samples. The quality of studies was overall good. It should be noted that some were highly specific with regard to the outcomes they measured (such as those that focused on rape), whereas others opted for vaguer measures of multifaceted adult sexual abuse. One advantage of the Bayesian approach we took was that the pervasive heterogeneity could be modelled and studied explicitly, allowing respectively for both a flexible overall estimate and an idea of how dispersed individual reports are. We were also able to identify natural subgroups of studies with relatively little variance of results, which we described individually in our analysis. To our knowledge, it is the first meta-analysis to estimate sexual abuse in groups of men with a mixed-diagnosis, with psychosis, with substance-misuse and with intellectual disablilities.

### Implications

The clinical implications of our study are a direct consequence of the high prevalence numbers reported in the studies we identified. Estimates of the number of cases of sexual violence that are missed in mental healthcare practices range between 70% and 90%.^85,86^ Seeing both that these estimates were derived mainly from research on female patients, and that the pervasiveness of dismissive beliefs about male sexual victimisation extend even to healthcare professionals, the number of undetected cases for men is likely to be even higher.^87^ At the same time, the strongest link in all types of trauma studied is that between sexual victimisation and psychopathology.^3^ The end result is a widespread cycle of underreporting, ineffectual detection, and, ultimately, undertreatment of trauma related to clinically important histories of sexual abuse in a number of psychiatric disorders. Various authors have already advocated guidelines for primary healthcare staff to collect relevant trauma history when treating patients.^88,89^

In practice, obtaining a history of interpersonal violence from this population in a sensitive and respectful manner has proven difficult.^90^ Stigma on both the patient and clinician side, shame, fear of being labelled a liar and self-blame remain barriers to overcome in responsibly detecting trauma.^91^ Our study further highlights the vital importance of responsibly obtaining a detailed history of trauma as a prerequisite for the effective treatment of patients with mental illness.

## Data Availability

Data and analysis code can be found on osf.io/4j8ct

## Supplementary material

For supplementary material accompanying this paper visit http://dx.doi.org/10.1192/bjo.2021.1069. click here to view supplementary material
