# Supplementary material for: Prevalence of adult sexual abuse in men with mental illness: Bayesian meta-analysis
Source: BJPsych Open. 2021 Dec 17;8(1):e16. doi: 10.1192/bjo.2021.1069 (PMC8715257; doi:10.1192/bjo.2021.1069)
Supplement: Supplementary file 1 [file S2056472421010693sup001.docx]

Prevalence of adult sexual abuse in men with mental illness: A Bayesian meta-analysis

Supplementary Materials

Keyword string used to search for studies across databases:

('sexual violence'/de OR 'sex trafficking'/de OR 'sexual assault'/de OR Rape/exp OR 'sexual abuse'/de OR 'sexual harassment'/exp OR 'sexual coercion'/de OR 'sexual exploitation'/de OR 'dating violence'/de OR 'sexual crime'/de OR (((sex* OR dating) NEAR/3 (violen* OR violation* OR traffick* OR assault* OR coerc* OR exploitat* OR abus* OR harass* OR bullying OR trauma* OR trading OR victim* OR non-consens* OR nonconsens* OR crime*)) OR rape):ab,ti) AND ('prevalence'/de OR epidemiology/de OR (prevalen* OR epidemiolog* OR ((rate OR rates OR Frequenc*) NEAR/3 (violence OR abuse))):ab,ti) AND ('mental health'/exp OR 'mental health care'/exp OR 'mental health center'/exp OR 'mental disease'/exp OR 'mental patient'/exp OR 'psychiatry'/exp OR 'psychiatric department'/de OR 'Diagnostic and Statistical Manual of Mental Disorders'/exp OR ((mental NEAR/3 (health* OR disease* OR patient* OR disorder*)) OR psychiatr* OR dual-diagnos* OR dsm OR ptsd OR ((posttraum* OR post-traum*) NEAR/3 stress) OR depress* OR borderline OR (personalit* NEAR/3 disorder*) OR bipolar* OR schizophreni* OR suicid* OR psychosis OR psychoses):ab,ti) NOT ((female/exp OR (women OR woman OR female*):ab,ti) NOT (male/exp OR (men OR man OR male*):ab,ti)) NOT ([Conference Abstract]/lim AND [1800-2017]/py) AND [English]/lim NOT ((juvenile/exp OR 'child abuse'/exp OR (child* OR boys OR girls):ab,ti) NOT (adult/exp OR (adult* OR men OR women):ab,ti))

| Supplementary Table 1. Studies included in the meta-analysis for each sample type | | | | | | | | | | |
| --- | --- | --- | --- | --- | --- | --- | --- | --- | --- | --- |
|  | **Author** | **Title** | **Sampling** | **Instrument** | **Implementation** | **Country** | **Quality** | **N (men)** | **Past year Sexual Abuse** | **Lifetime** |
| Mixed-diagnosis | Goodman 2001 | Recent victimization in women and men with severe Mental illness: prevalence and correlates | Mixed | CTS | Face-to-face | US | Good | 461 | 7.6 | 24.5 |
| Mixed-diagnosis | Anderson 2016 | Childhood maltreatment and adulthood domestic and sexual Violence victimisation among people with severe mental illness | Random | CTQ | Face-to-face | UK | Good | 181 | — | 21.6 |
| Mixed-diagnosis | Yen 2002 | Traumatic exposure and posttraumatic stress disorder in borderline, Schizotypal, avoidant, and obsessive-compulsive personality disorders: Findings from the collaborative longitudinal personality disorders study | Convenience | SCID trauma addendum | Face-to-face | US | Low | 244 | — | 19.3 |
| Mixed-diagnosis | De Oliveira 2012 | Factors associated with self-report of sexual violence Against men and women with mental disorders in brazil | Random | Original questions | Face to face | Brazil | Good | 1198 | — | 12.5 |
| Mixed-diagnosis | De Waal 2017 | Gender differences in characteristics of physical and sexual victimization in  patients with dual diagnosis: a cross-sectional study | Random | Safety monitor (Veiligheidsmonitor) | Face to face | Netherlands | Good | 171 | 4.1 | — |
| Mixed-diagnosis | Coverdale 2000 | Sexual and physical abuse of chronically ill psychiatric outpatients compared with a matched sample of medical outpatients | Unclear | Clinical interview | Face to face | New Zealand | Good | 92 | — | 26.1 |
| Mixed-diagnosis | Hutchings 1993 | Sexual assault history In a community mental health Center clinical population | Unclear | Original questions | Self-administered | US | Low | 41 | — | 24 |
| Mixed-diagnosis | Kamperman 2014 | Criminal victimisation in people with severe mental Illness: a multi-site prevalence and incidence survey in The Netherlands | Random | Dutch crime and victimisation survey | Face-to-face | Netherlands | Good | 608 | 3 | — |
| Mixed-diagnosis | Khalifeh 2015 | Domestic and sexual violence against patients with Severe mental illness | Random | Crime survey for England and wales (CSEW) | Self-completed | UK | Good | 157 | 3.2 | 22.9 |
| Mixed-diagnosis | Lipschitz 1996 | Prevalence and characteristics Of physical and sexual abuse Among psychiatric outpatients | Random | Traumatic events questionnaire (TEQ) | Self-completed | US | Good | 34 | — | 14.7 |
| Mixed-diagnosis | Dammeyr 2018 | A national survey on violence and Discrimination among people with Disabilities | Random | Original questions | Online questionnaire | Denmark | Good | 493 | 2 | — |
| Mixed-diagnosis | Shack 2004 | Prior history of physical and Sexual abuse among the Psychiatric inpatient population: A comparison of males and females | Convenience | Clinical interview | Face-to-face | US | Low | 160 | — | 25 |
| Mixed-diagnosis | Teplin 2005 | Crime victimization in adults with severe mental illness: Comparison with the national crime victimization survey | Random | NCSV | Face-to-face | US | Good | 483 | 0.8 | — |
| Mixed-diagnosis | Mueser 1998 | Trauma and posttraumatic stress disorder in severe mental illness | Convenience | Trauma History questionnaire (THQ) | Face-to-face | US | Good | 122 | — | 25.9 |
| Mixed-diagnosis | Villano 2007 | Prevalence and correlates of posttraumatic stress disorder and chronic Severe pain in psychiatric outpatients | Convenience | Stressful Life events inventory (SLEI) | Face-to-face | US | Good | 175 | — | 12.6 |
| Intellectual disability | Llario 2019 | Prevalence and sequelae of self-reported and other reported Sexual abuse in adults with intellectual disability | Unclear | Clinical interview | Self-report/documented | Spain | Good | 180 | — | 29.4 |
| Intellectual disability | Pan 2007 | Prevalence of sexual abuse of people with intellectual Disabilities in Taiwan | Probability cluster sampling | Clinical interview | Face-to-face/family interviews | Taiwan | Good | 190 | — | 4.2 |
| Intellectual disability | Platt 2017 | The role of gender in Violence experienced By adults with Developmental Disabilities | Mixed | Assisted computer interview | Anonymous self-report | US | Good | 172 | — | 7.1 |
| Psychosis | Amir 2012 | Prevalence and correlates of physical and sexual assault history in patients With schizophrenia | Convenience | Trauma assessment for adults (TAA) | Face-to-face | Egypt | Good | 61 | — | 26.2 |
| Psychosis | Calhoun 2007 | Interpersonal trauma, war zone exposure, and posttraumatic stress Disorder among veterans with schizophrenia | Convenience | SAEQ | Face-to-face | US | Good | 165 | 10.3 | 26.7 |
| Psychosis | Bengtsson-tops 2012 | Victimization in individuals suffering from psychosis: A Swedish cross-sectional study | Random | Composite abuse scale (CAS) | Face-to-face | Sweden | Good | 75 | 6.7 | 13.3 |
| PTSD | Lapp 2005 | Lifetime sexual and physical victimization among male veterans With combat-related post-traumatic stress disorder | Convenience | CTS | Face-to-face | US | Good | 133 | 6.02 | 47.3 |
| PTSD | Clancy 2006 | Lifetime trauma exposure in veterans With military-related posttraumatic stress disorder: Association with current symptomatology | Convenience | TLEQ | Face-to-face | US | Low | 422 | — | 5.9 |
| Substance abuse | Tiet 2006 | Recent sexual abuse, physical abuse, and suicide attempts among male Veterans seeking psychiatric treatment | Unclear | Addiction severity index | Face-to-face | US | Good | 33236 | — | 8.4 |
| Substance abuse | Moncrieff 1996 | Sexual abuse in people with alcohol problems. A study of the prevalence of Sexual abuse and its relationship to drinking behaviour | Unclear | Original questions | Face-to-face | UK | Low | 89 | — | 23.6 |
| Substance abuse | Afful 2010 | Exposure to trauma: a comparison of cocaine-dependent cases and a Community-matched sample | Unclear | Clinical interview | Face-to-face | US | Good | 216 | — | 11.6 |
| Substance abuse | Bone 2018 | Prevalence of sexual violence and its association With depression among male and female patients with risky Drug use in urban federally qualified health centers | Random | Original questions | Self-administered through tablet | US | Low | 210 | — | 24.8 |
| Substance abuse | Armstrong 2014 | Suicidal ideation and attempts among men who inject drugs In Delhi, India: psychological and social risk factors | Probability | Clinical interview | Face-to-face | India | Good | 420 | — | 15.8 |
| Substance abuse | Bonin 2000 | Drinking away the hurt: the nature and prevalence Of PTSD in substance abuse patients attending A community-based treatment program | Convenience | Original questions | Face-to-face | Canada | Low | 61 | — | 8.2 |
| Substance abuse | Barry 2011 | Exploring relations among traumatic, posttraumatic, and physical Pain experiences in methadone-maintained patients | Convenience | Life experiences checklist (LEC) | Face-to-face | US | Low | 85 | — | 16.5 |
| Substance abuse | Braitstein 2003 | Sexual violence among a cohort of injection drug users | Convenience | Original questions | Face-to-face | Canada | Low | 932 | — | 18.6 |
| Substance abuse | Branstetter 2008 | A history of sexual, emotional, or physical abuse predicts adjustment During opioid maintenance treatment | Convenience | ASI | Face-to-face | US | Low | 191 | — | 4 |
| Substance abuse | Dansky 1996 | Victimization and PTSD in individuals with Substance use disorders: gender and racial Differences | Convenience | Clinical interview | Face-to-face | US | Low | 34 | — | 20.6 |
| Substance abuse | Dworkin 2017 | The unique associations of sexual assault and intimate partner violence with PTSD symptom clusters in a traumatized substance-abusing sample | Convenience | National women’s study PTSD module | Face to face | US | Low | 112 | — | 33.9 |
| Substance abuse | Fiorentine 1999 | Drug treatment outcomes: investigating the Long-term effects of sexual and physical abuse Histories | Convenience | Clinical interview | Face to face | US | Good | 117 | — | 13.6 |
| Substance abuse | Daigre 2015 | History of sexual, emotional or physical abuse and psychiatric Comorbidity in substance-dependent patients | Convenience | European addiction severity index | Face-to-face | Spain | Good | 387 | — | 6.7 |
| Substance abuse | Pearce 2008 | The cedar project: historical trauma, sexual abuse and HIV risk among Young aboriginal people who use injection and non-injection drugs in Two Canadian cities | Convenience | Clinical interview | Face to face | Canada | Low | 281 | — | 28.8 |
| Substance abuse | Guimaraes 2017 | Gender differences in patterns of drug use And sexual risky behaviour among crack Cocaine users in central brazil | Convenience | Clinical interview | Face to face | Brazil | Low | 914 | — | 8.2 |
| Substance abuse | Jakubczyk 2014 | History of sexual abuse and suicide attempts in alcohol-dependent patients | Convenience | Substance abuse outcomes module | Face to face | Poland | Low | 284 | — | 7.4 |
| Substance abuse | Mckeganey 2017 | Physical and sexual abuse among drug users Contacting drug treatment services in Scotland | Convenience | Clinical interview | Face-to-face | Scotland | Low | 715 | — | 6.9 |
| Mixed intimate partner | Tham 1995 | A survey of domestic violence and other forms of abuse | Convenience | Original questions | Face-to-face | UK | Low | 91 | — | 4.4 |
| Mixed intimate partner | Heru 2006 | Prevalence and severity of intimate partner violence and associations with family functioning and alcohol abuse in psychiatric patients with suicidal intent | Convenience | Cts2 | Face-to-face | US | Good | 44 | 32 | — |
| Mixed intimate partner | Chang 2011 | Partner violence screening in mental health | Convenience | AAS | Face-to-face | US | Good | 158 | 3 | 4 |
| Mixed intimate partner | Khalifeh 2015 | Domestic and sexual violence against patients with Severe mental illness | Random | CSEW | Face-to-face | UK | Good | 170 | — | 4.1 |
| Mixed intimate partner | Ruiz-Perez 2018 | Intimate partner violence and mental disorders: co-occurrence and gender Differences in a large cross-sectional population based study in Spain | Random | Original questions | Face-to-face | Spain | Good | 407 | 2.2 | — |
| Mixed intimate partner | Shack 2004 | Prior history of physical and Sexual abuse among the Psychiatric inpatient population: A comparison of males and females | Convenience | Original questions | Face-to-face | US | Low | 160 | — | 7.5 |
| Substance abuse intimate partner | Kalokhe 2012 | Intimate partner violence among HIV-infected Crack cocaine users | Convenience | STAT | Face-to-face | US | Low | 170 | — | 6 |

| *Supplementary Table 2. Comparing random and fixed effect models based on the Leave-One-Out (LOO) and Widely Applicable Information Criterion (WAIC). Lower values indicate better model fit.* | | | | |
| --- | --- | --- | --- | --- |
|  | **LOO** | **LOO Std. Error** | **WAIC** | **WAIC Std. Error** |
| **Past year** | | | | |
| Random Effects | 79.479 | 5.061 | 68.623 | 3.108 |
| Fixed Effects | 152.962 | 40.204 | 152.796 | 40.184 |
| **Adulthood** | | | | |
| Random Effects | 296.439 | 9.798 | 263.393 | 8.250 |
| Fixed Effects | 1148.927 | 214.247 | 1181.196 | 234.228 |

Supplementary Figure 1. Posterior predictive checks for the past year and adulthood models. In dark blue is distribution of empirical data (study prevalences), light blue is simulated data from the model (estimated prevalences).


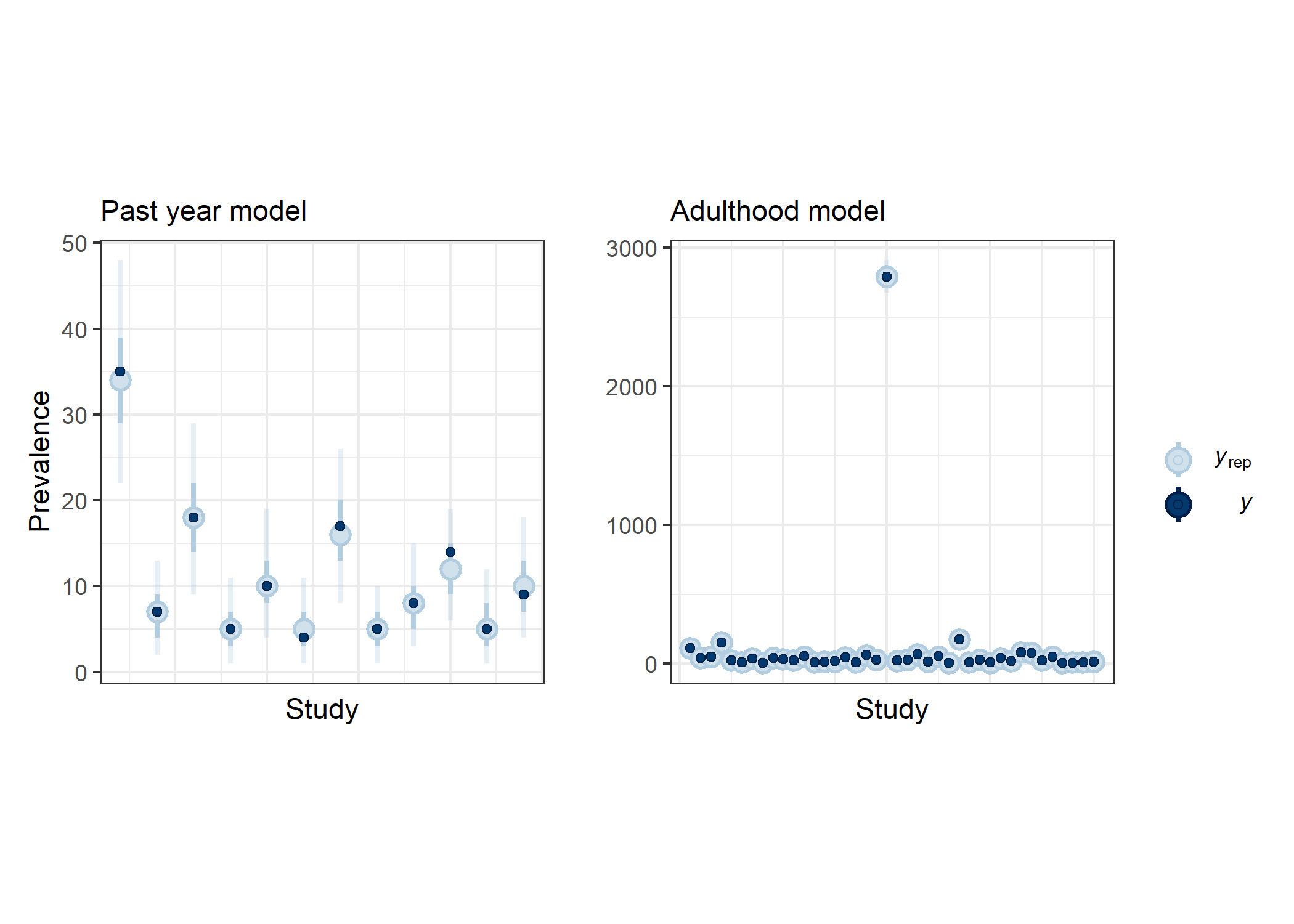


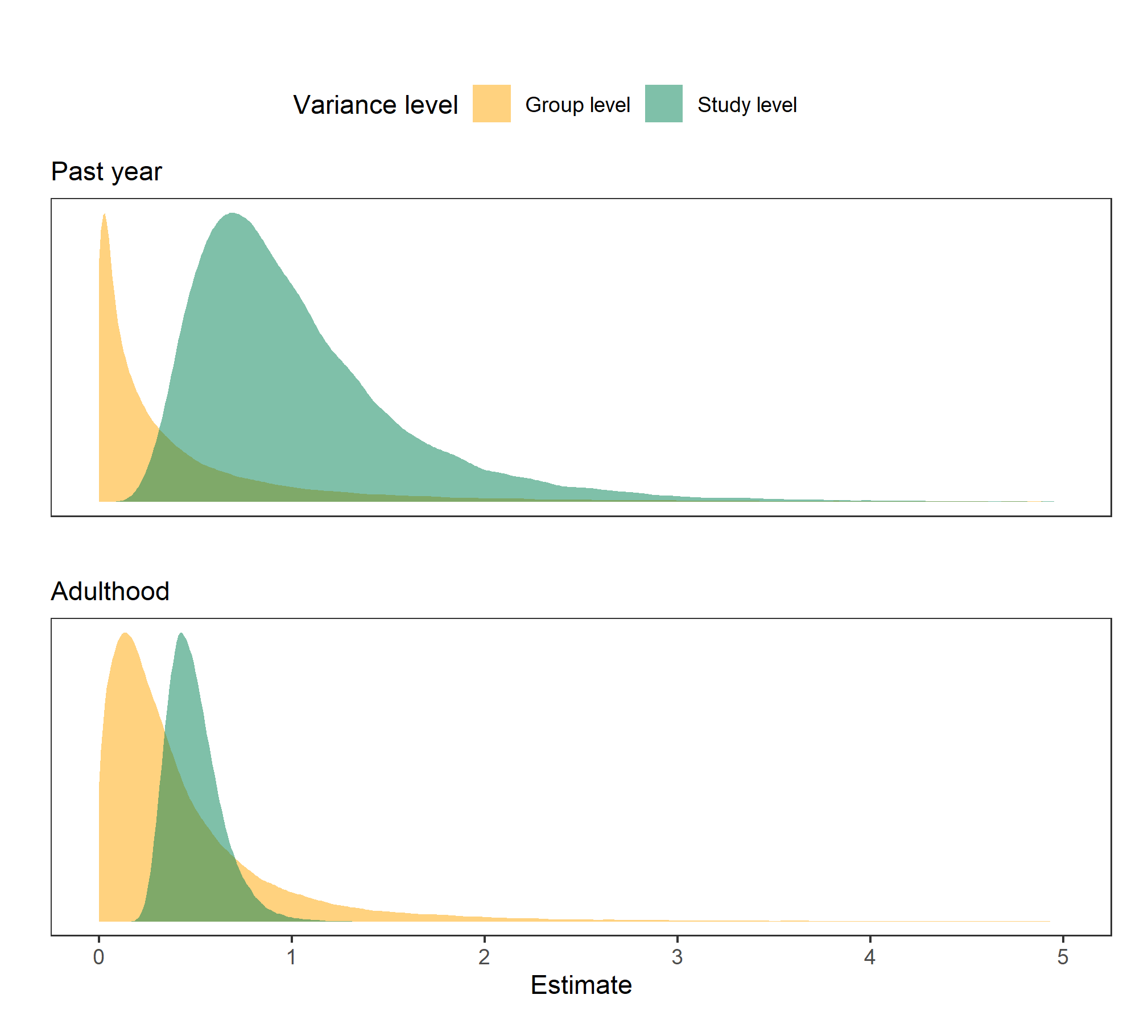


Supplementary Figure 2. Posterior distribution of between-study variance coefficient for the past year and adulthood sexual abuse models. Higher values indicate more variance between studies or groups.


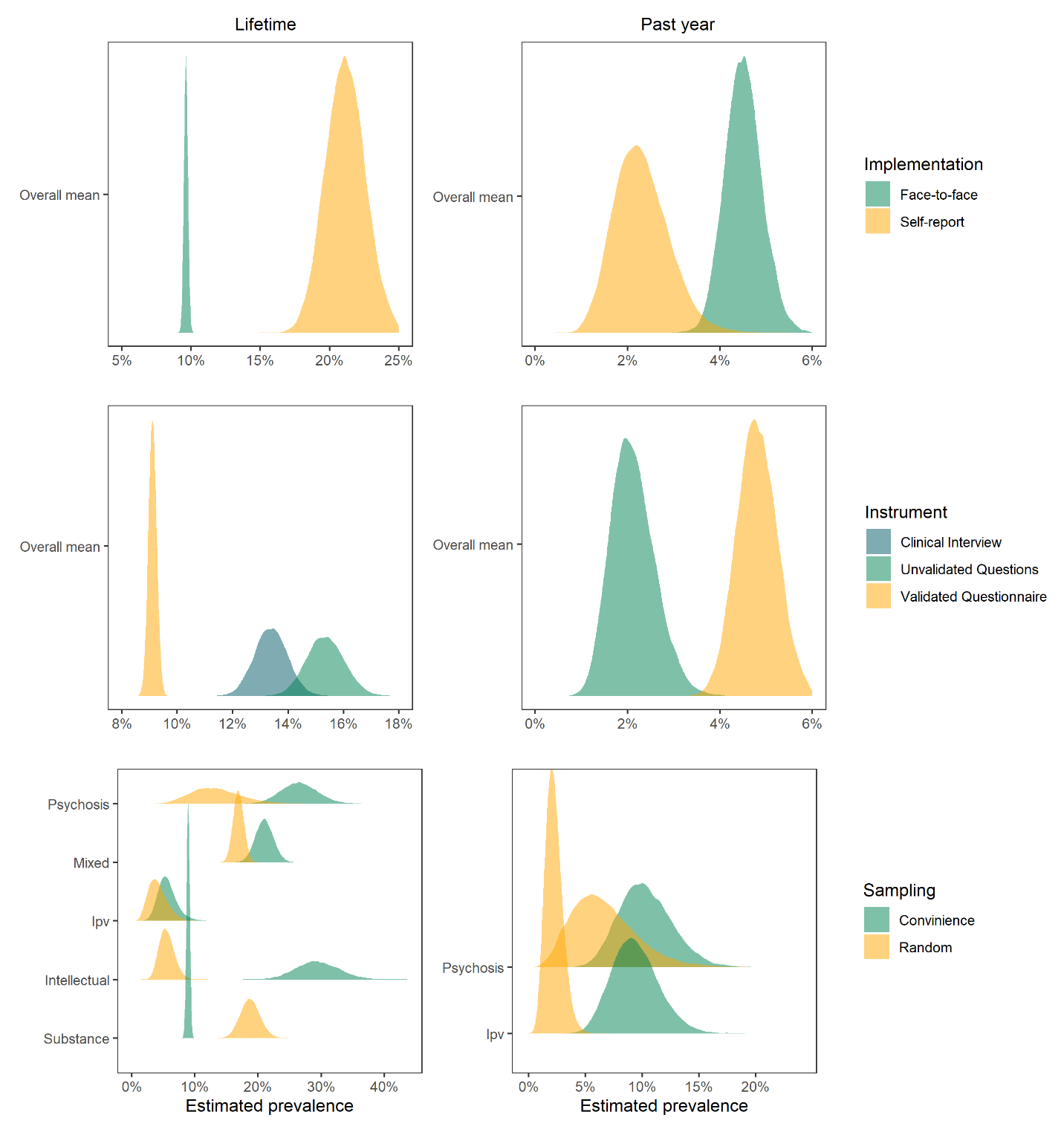


Supplementary Figure 3. Comparisons of reported prevalence by differences in study design.
